# Predictive Value of Triglyceride/HDL‐C Ratio for Unplanned Coronary Revascularization in Elderly Patients With Type 2 Diabetes and Coronary Artery Disease: A Retrospective Cohort Study

**DOI:** 10.1002/agm2.70058

**Published:** 2025-12-12

**Authors:** Wei Zhu, Yin Zhang, Pan Gao

**Affiliations:** ^1^ Department of Geriatrics Southwest Hospital Affiliated to the Third Military Medical University (Army Medical University) Chongqing China

**Keywords:** coronary artery disease, elderly, triglyceride‐to‐HDL cholesterol ratio (TG/HDL‐C ratio), type 2 diabetes mellitus, unplanned coronary revascularization

## Abstract

**Objectives:**

The triglyceride‐to‐high‐density lipoprotein cholesterol (TG/HDL‐C) ratio reflects atherogenic dyslipidemia and insulin resistance. Its predictive value for unplanned coronary revascularization in elderly patients with type 2 diabetes mellitus (T2DM) and coronary artery disease (CAD) is unclear.

**Methods:**

We retrospectively analyzed 1796 patients aged ≥ 60 years with T2DM and angiographically confirmed CAD from January 2008 to November 2021. The primary endpoint was unplanned coronary revascularization, defined as revascularization performed because of angina symptoms, new ischemic changes on ECG, or signs of reversible myocardial ischemia on noninvasive imaging. TG/HDL‐C ratio was evaluated by tertiles in Kaplan–Meier analysis and as a continuous variable in Cox models: Model 1 (unadjusted), Model 2 (age, gender, smoking), and Model 3 (further adjusted for lipid, metabolic, renal, and angiographic covariates). Restricted cubic spline (RCS) analysis and prespecified subgroup analyses were performed.

**Results:**

During a median follow‐up of 1175 days (interquartile range, 597–1986), unplanned revascularization occurred in 309 patients (17.2%). In the fully adjusted model, TG/HDL‐C ratio remained independently associated with increased risk (per 1‐unit increase: hazard ratio [HR] 1.029, 95% confidence interval [CI] 1.007–1.051, *p* = 0.011; per standard deviation increase: HR 1.158, 95% CI 1.039–1.290, *p* = 0.008). Compared with the lowest tertile, the highest tertile showed a significantly higher risk of unplanned revascularization (HR = 1.646, 95% CI = 1.133–2.393, *p* < 0.010). The RCS analysis demonstrated a relatively flat risk below approximately 3.427, with a progressive increase thereafter (*p* for overall association = 0.006; *p* for nonlinearity = 0.045). Subgroup analyses showed no statistically significant interactions (all *p* for interaction > 0.05), and the direction of association was consistent across predefined clinical strata.

**Conclusion:**

Higher TG/HDL‐C ratio independently predicted unplanned revascularization in elderly patients with T2DM and CAD. This simple, widely available lipid parameter may aid long‐term risk stratification, but prospective multicenter studies are needed for validation.

## Graphical abstract

Elevated TG/HDL‐C ratio independently predicted unplanned coronary revascularization in elderly patients with type 2 diabetes and CAD, highlighting its value as a simple and accessible biomarker for long‐term risk stratification.

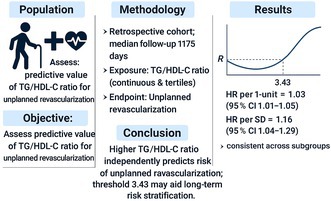

## 1 Introduction

Cardiovascular disease (CVD) remains the leading cause of morbidity and mortality worldwide, particularly among elderly individuals and those with T2DM and CAD [1, 2, 3]. Accurate identification of high‐risk patients is essential for optimizing secondary prevention strategies and improving long‐term outcomes [4, 5, 6].

The TG/HDL‐C ratio is a simple, inexpensive lipid parameter that reflects atherogenic dyslipidemia and insulin resistance [7, 8, 9]. An elevated TG/HDL‐C ratio has been associated with an increased risk of incident cardiovascular events in diverse populations, including those with metabolic syndrome, diabetes, and acute coronary syndromes [10, 11]. It has also been shown to predict repeat revascularization after first‐time PCI in acute coronary syndrome [12]. However, that evidence was derived from relatively younger ACS cohorts, and its prognostic significance in elderly patients with T2DM and established CAD, particularly for long‐term, clinically driven unplanned revascularization, remains unclear [13].

Unplanned revascularization, which often reflects disease progression or recurrent ischemia, represents a clinically relevant endpoint that impacts patient quality of life, healthcare costs, and prognosis [14, 15]. Identifying predictors of unplanned revascularization may provide an opportunity for early intervention and targeted therapy [16, 17].

Therefore, the present study aimed to investigate the association between the TG/HDL‐C ratio and the risk of unplanned coronary revascularization in elderly patients with T2DM and CAD. We further applied RCS analyses to evaluate potential nonlinear relationships and performed subgroup analyses to assess the robustness of the association across clinically relevant strata.

## 2 Methods

### 2.1 Study Design and Population

This retrospective cohort study included elderly patients (aged ≥ 60 years) with T2DM and angiographically confirmed CAD who underwent coronary angiography at Southwest Hospital between January 2008 and November 2021. Patients were excluded if they lacked baseline TG or HDL‐C measurements, had incomplete clinical data, did not undergo coronary angiography at our institution, or had no follow‐up information after enrollment. After applying these criteria, 1796 patients were included in the final analysis.

The study protocol was approved by the Ethics Committee of Southwest Hospital (Approval No. (B) KY2021189), affiliated with the Third Military Medical University (Army Medical University), and was conducted in accordance with the Declaration of Helsinki. Given its retrospective design, the requirement for informed consent was waived.

### 2.2 Data Collection and Definitions

Baseline demographic, clinical, laboratory, and angiographic data were extracted from the institutional electronic medical record system, including age, sex, body mass index, smoking status, comorbidities (hypertension, myocardial infarction history, atrial fibrillation, chronic heart failure, chronic obstructive pulmonary disease, hyperlipidemia, carcinoma), laboratory parameters (fasting blood glucose, glycated hemoglobin [HbA1c], lipid profiles, serum albumin, creatinine, white blood cell count, hemoglobin), and angiographic findings (multivessel disease, Gensini score). Discharge medications (antiplatelet agents, lipid‐lowering therapy, β‐blockers, insulin) were also recorded. The TG/HDL‐C ratio was calculated by dividing fasting TG by HDL‐C, and patients were categorized into tertiles (T1–T3) based on its distribution.

### 2.3 Follow‐Up and Outcome

Participants were followed from the date of the index percutaneous coronary intervention (PCI) until the occurrence of the primary endpoint, death, or the end of the study period (October 31, 2022), whichever occurred first. Follow‐up data were obtained from outpatient visits, rehospitalization records, and telephone interviews. The primary endpoint was unplanned coronary revascularization, defined as revascularization performed because of angina symptoms, new ischemic changes on ECG, or signs of reversible myocardial ischemia on noninvasive imaging [18]. Events were ascertained through the review of complete hospital records in accordance with standardized diagnostic and procedural criteria.

### 2.4 Statistical Analysis

Continuous variables are presented as mean ± standard deviation (SD) or median (interquartile range, IQR) and were compared using one‐way analysis of variance (ANOVA) or the Kruskal–Wallis test, as appropriate. Categorical variables are expressed as counts (percentages) and compared using the chi‐square test or Fisher's exact test.

Missing covariate data were handled using multiple imputation with the random forest method under the missing at random assumption. The association between TG/HDL‐C ratio and the risk of unplanned revascularization was evaluated using Cox proportional hazards regression models, with TG/HDL‐C ratio analyzed both as a continuous variable and in tertiles (T1–T3). Three models were constructed: Model 1, unadjusted; Model 2, adjusted for gender, age, and smoking status; Model 3, additionally adjusted for HDL‐C, albumin, LDL‐C, total cholesterol, white blood cell count, creatinine, fasting blood glucose, HbA1c, and Gensini score. Kaplan–Meier survival curves were generated for unadjusted and Model 3‐adjusted estimates, with differences assessed by the log‐rank test. Dose–response relationships were explored using RCS regression based on Model 3, with the median TG/HDL‐C ratio as the reference. Subgroup analyses were performed for prespecified clinical categories. For each subgroup, separate Cox models were fitted adjusting for the Model 3 covariates except the stratifying factor. Interaction was tested by adding a multiplicative cross product term to Model 3 in the overall cohort. All statistical analyses were conducted using R software (version 4.4.1, Vienna, Austria). A two‐sided *p* value < 0.05 was considered statistically significant.

## 3 Results

### 3.1 Baseline Characteristics

Table 1 presents the baseline characteristics of the study population categorized by the occurrence of unplanned revascularization. The median follow‐up duration was 1175 days (IQR: 597–1986), with the non‐revascularization group having a longer median follow‐up of 1224 days (IQR: 666–2037) compared with 771 days (IQR: 390–1604) for the revascularization group. The median age was 73 years (IQR: 68–78), and 38.4% were female.

**TABLE 1:** Baseline clinical characteristics according to unplanned revascularization status.

Variable	Total (*n* = 1796)	No unplanned revascularization (*n* = 1487)	Unplanned revascularization (*n* = 309)	*p*
Age, years	73.0 (68.0, 78.0)	73.0 (68.0, 78.0)	73.0 (68.0, 77.0)	0.449
Gender, %				
Female	690 (38.4)	603 (40.6)	87 (28.2)	
Male	1106 (61.6)	884 (59.4)	222 (71.8)	< 0.001
Current smoker, %	504 (28.1)	381 (25.6)	123 (39.8)	< 0.001
SBP, mmHg	133.5 (120.0, 146.0)	134.0 (120.0, 146.0)	132.0 (120.0, 146.0)	0.778
DBP, mmHg	78.0 (70.0, 82.0)	78.0 (70.0, 82.0)	78.0 (70.0, 82.0)	0.790
BMI, kg/m^2^	24.8 (22.9, 26.9)	24.8 (22.9, 27.0)	24.8 (23.0, 26.6)	0.226
HGB, g/L	132.0 (121.0, 142.0)	132.0 (121.0, 142.0)	132.0 (120.5, 143.0)	0.964
WBC, × 10^9^	6.3 (5.3, 7.7)	6.2 (5.3, 7.6)	6.6 (5.4, 8.2)	0.018
Alb, g/L	39.0 (36.8, 41.1)	39.1 (36.9, 41.2)	38.4 (36.2, 40.6)	0.005
ALT, IU/L	19.9 (14.6, 28.9)	19.8 (14.4, 28.5)	20.0 (14.9, 30.0)	0.318
LDL‐C, mmol/L	2.5 (2.0, 3.1)	2.5 (2.0, 3.1)	2.6 (2.1, 3.2)	0.008
TG, mmol/L	1.4 (1.1, 2.1)	1.4 (1.1, 2.1)	1.5 (1.1, 2.2)	0.037
HDL‐C, mmol/L	1.0 (0.9, 1.2)	1.0 (0.9, 1.2)	1.0 (0.9, 1.1)	0.240
Tch, mmol/L	4.1 (3.4, 4.9)	4.0 (3.4, 4.9)	4.3 (3.6, 5.0)	0.006
Cr, μmol/L	75.0 (62.6, 90.0)	75.0 (62.0, 88.9)	77.0 (65.0, 93.1)	0.013
FBG, mmol/L	6.7 (5.5, 9.1)	6.6 (5.5, 8.9)	7.2 (5.7, 9.9)	0.001
HbA1c, %	6.8 (6.3, 7.8)	6.7 (6.2, 7.8)	6.9 (6.3, 7.9)	0.058
Insulin use, %	311 (17.3)	238 (16.0)	73 (23.6)	0.002
MI history, %	317 (17.7)	219 (14.7)	98 (31.7)	< 0.001
PCI history, %	1187 (66.1)	914 (61.5)	273 (88.3)	< 0.001
Multivessel disease, % < 0.001				< 0.001
1‐vessel disease	1228 (68.4)	1102 (74.1)	126 (40.8)	
2‐vessel disease	354 (19.7)	256 (17.2)	98 (31.7)	
3‐vessel disease	214 (11.9)	129 (8.7)	85 (27.5)	
Gensini score	33.5 (26.5, 52.5)	31.5 (26.0, 46.0)	56.5 (37.0, 86.5)	< 0.001
TG/HDL‐C ratio	3.3 (2.2, 5.1)	3.3 (2.2, 5.0)	3.5 (2.4, 5.5)	0.026

*Note:* Normally distributed data of continuous variables were determined using the Shapiro–Wilk normality test. The Wilcoxon rank‐sum test compared between‐group differences due to non‐normal distribution. The Chi‐square test assessed categorical variables between‐group differences.

Abbreviations: Alb, albumin; ALT, alanine aminotransferase; BMI, body mass index; Cr, serum creatinine; DBP, diastolic blood pressure; FBG, fasting blood glucose; HbA1c, glycosylated hemoglobin; HDL‐C, high‐density lipoprotein cholesterol; HGB, hemoglobin; LDL‐C, low‐density lipoprotein cholesterol; MI, myocardial infarction; PCI, percutaneous coronary intervention; SBP, systolic blood pressure; Tch, total cholesterol; TG, triglyceride; WBC, white blood cell count.

Compared with patients without unplanned revascularization, those who experienced revascularization were more frequently male, current smokers, and insulin users, and had higher proportions of prior myocardial infarction, prior PCI, and multivessel coronary artery disease (all *p* < 0.05). Laboratory findings revealed significantly higher levels of neutrophils, WBC count, LDL‐C, total cholesterol, triglycerides, creatinine, and fasting blood glucose, as well as lower serum albumin concentrations in the revascularization group (all *p* < 0.05). The Gensini score and TG/HDL‐C ratio were also significantly higher among patients with events (both *p* < 0.05). No significant differences were observed for age, BMI, blood pressure, hemoglobin, lymphocyte count, or HbA1c (all *p* > 0.05).

### 3.2 Association Between TG/HDL‐C Ratio and Risk of Unplanned Revascularization

In the univariable Cox analysis (Table 2), a higher TG/HDL‐C ratio was significantly associated with an increased risk of unplanned revascularization (HR per 1‐unit increase, 1.020; 95% CI, 1.004–1.036; *p* = 0.017). In the multivariable model adjusting for potential confounders, the TG/HDL‐C ratio remained an independent predictor (HR, 1.029; 95% CI, 1.007–1.051; *p* = 0.011).

**TABLE 2:** Univariate and multivariate Cox regression analyses of TG/HDL‐C ratio for unplanned revascularization.

Variable	Univariate		Multivariate	
	HR (95% CI)	*p*	HR (95% CI)	*p*
TG/HDL‐C ratio	1.020 (1.004–1.036)	0.017	1.029 (1.007–1.051)	0.011
Gender				
Female	Reference		Reference	
Male	1.601 (1.249–2.053)	< 0.001	1.356 (1.006–1.827)	0.046
Age	0.986 (0.970–1.002)	0.088	0.993 (0.975–1.011)	0.435
Smoking				
Non‐smoker	Reference		Reference	
Current smoker	1.610 (1.281–2.025)	< 0.001	1.302 (0.995–1.705)	0.056
PCI history	3.935 (2.779–5.574)	< 0.001	2.341 (1.626–3.370)	< 0.001
MI history	2.442 (1.919–3.107)	< 0.001	1.255 (0.953–1.652)	0.106
Multivessel disease				
1‐vessel	Reference		Reference	
2‐vessel	3.187 (2.443–4.159)	< 0.001	1.782 (1.325–2.396)	< 0.001
3‐vessel	5.407 (4.101–7.130)	< 0.001	2.176 (1.506–3.145)	< 0.001
Insulin use	1.577 (1.213–2.051)	0.001	1.187 (0.869–1.622)	0.282
HDL‐C	0.816 (0.522–1.275)	0.373	1.976 (1.106–3.529)	0.022
Alb	0.933 (0.902–0.964)	< 0.001	0.965 (0.933–1.000)	0.048
LDL‐C	1.165 (1.030–1.318)	0.016	1.307 (0.935–1.826)	0.118
Tch	1.116 (1.025–1.216)	0.013	0.853 (0.660–1.103)	0.228
WBC	1.048 (0.997–1.101)	0.069	0.950 (0.899–1.004)	0.069
Cr	1.001 (0.999–1.003)	0.287	1.000 (0.998–1.003)	0.881
FBG	1.044 (1.023–1.066)	< 0.001	1.000 (0.972–1.029)	0.987
HbA1c	1.105 (1.033–1.182)	0.004	1.048 (0.957–1.148)	0.313
Gensini score	1.018 (1.016–1.021)	< 0.001	1.011 (1.007–1.015)	< 0.001

We further assessed the effect of TG/HDL‐C ratio per 1‐unit and per 1‐standard deviation (SD) increase across models (Table 3). The association remained consistent and statistically significant from unadjusted to fully adjusted models. In the fully adjusted model, each 1‐unit increase was associated with a 2.9% higher risk (HR, 1.029; 95% CI, 1.007–1.051; *p* = 0.011), and each 1‐SD increase was associated with a 15.8% higher risk (HR, 1.158; 95% CI, 1.039–1.290; *p* = 0.008).

**TABLE 3:** Hazard ratios (HR) for unplanned revascularization according to TG/HDL‐C ratio in different models.

Exposure	Model 1		Model 2		Model 3	
	HR (95% CI)	*p*	HR (95% CI)	*p*	HR (95% CI)	*p*
TG/HDL‐C ratio (per 1 unit increase)	1.020 (1.004–1.036)	0.017	1.017 (1.000–1.033)	0.049	1.029 (1.007–1.051)	0.011
TG/HDL‐C ratio (per 1 SD increase)	1.100 (1.017–1.189)	0.017	1.083 (1.001–1.172)	0.048	1.158 (1.039–1.290)	0.008

*Note:* Model 1: Unadjusted. Model 2: Adjusted for gender, age, and smoking. Model 3: Additionally adjusted for HDL‐C, albumin, LDL‐C, Tch, WBC, creatinine, fasting blood glucose, HbA1c, and Gensini score.

**FIGURE 1:**
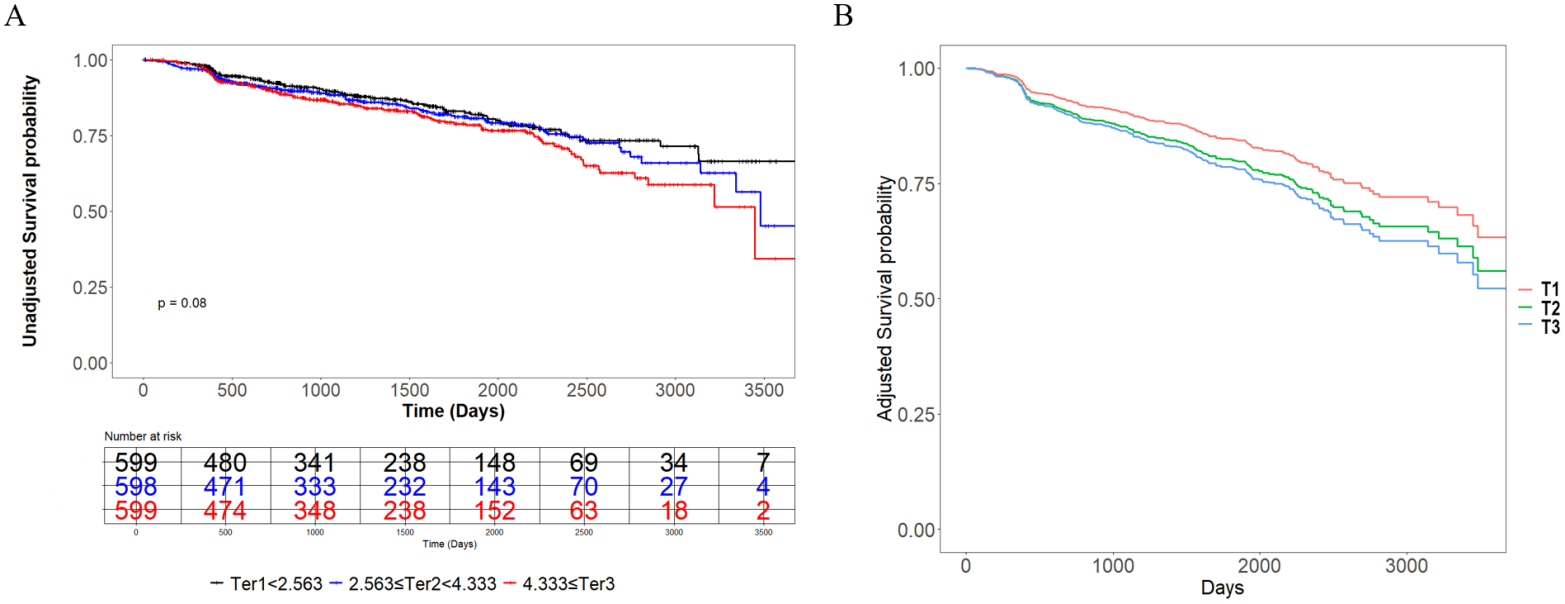
Kaplan–Meier analysis of TG/HDL‐C ratio and unplanned revascularization. Kaplan–Meier curves showing the cumulative incidence of unplanned revascularization according to TG/HDL‐C ratio tertiles. (A) Unadjusted model. (B) Adjusted for gender, age, smoking status, history of myocardial infarction, prior PCI, insulin use, Multivessel disease, HDL‐C, albumin, LDL‐C, total cholesterol, white blood cell count, creatinine, fasting blood glucose, HbA1c, and Gensini score.

Kaplan–Meier survival curves stratified by TG/HDL‐C tertiles (Figure 1) showed no significant difference in the unadjusted model (Figure 1A, log‐rank *p* = 0.080). After adjustment for the same covariates as in Model 3 (Figure 1B), the curves demonstrated a clear stepwise increase in the cumulative incidence of unplanned revascularization from T1 to T3 (log‐rank *p* < 0.001). This shift from nonsignificant to significant association likely reflects the control of confounding factors, such as demographic characteristics, comorbidities, and laboratory parameters, which may have otherwise masked the true relationship in the unadjusted analysis.

### 3.3 Dose–Response Relationship

**FIGURE 2:**
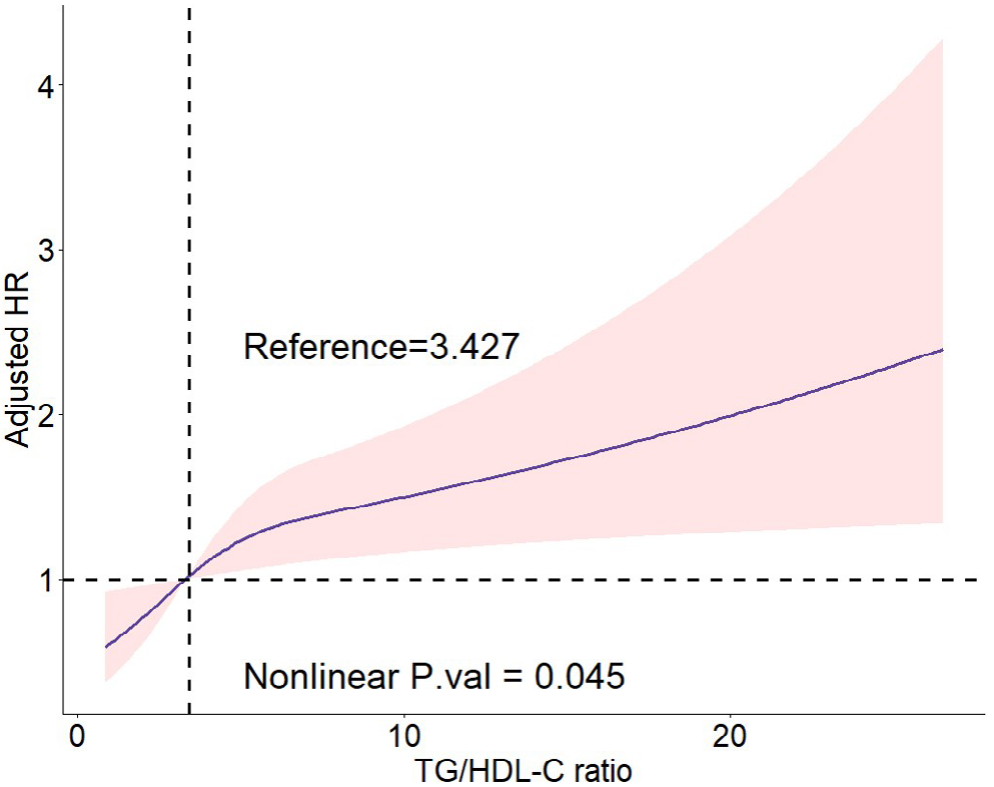
Association between TG/HDL‐C ratio and unplanned revascularization. Restricted cubic spline plot showing adjusted hazard ratios (HRs) and 95% *CIs* for TG/HDL‐C ratio in relation to unplanned revascularization (Model 3). HR = 1 corresponds to a TG/HDL‐C ratio of 3.427.

The restricted cubic spline (RCS) analysis based on the fully adjusted Cox proportional hazards model (Model 3; Figure 2) showed a significant overall association between the TG/HDL‐C ratio and the hazard of unplanned revascularization (*p* for overall association = 0.006) and a statistically significant nonlinear component (*p* for nonlinearity = 0.045). The curve crossed the null line (HR = 1.000) at a TG/HDL‐C ratio of 3.427, which was used as the reference point in the RCS analysis. Below this point, HRs were close to unity, whereas higher values were associated with progressively greater risk in a mildly nonlinear fashion.

### 3.4 Subgroup Analyses

**FIGURE 3:**
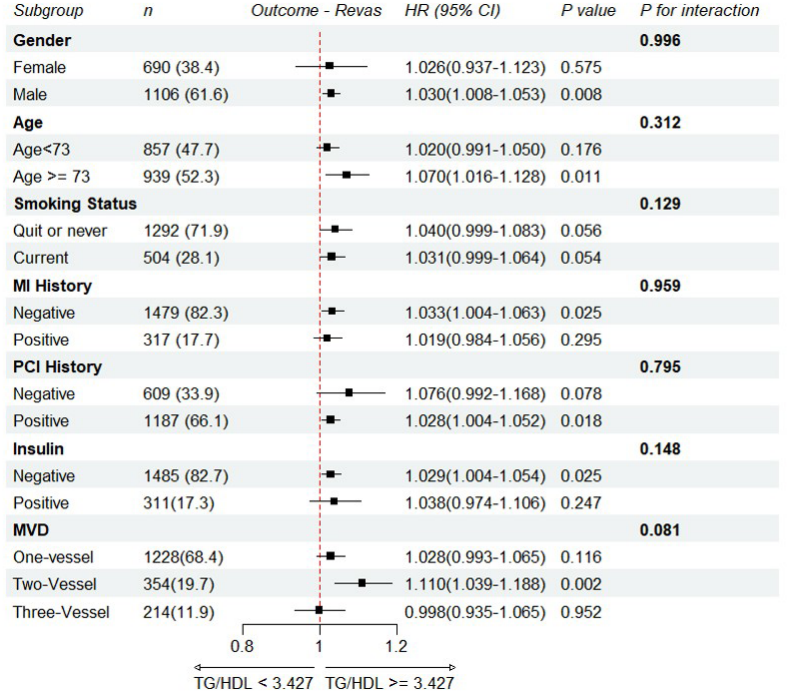
Subgroup analyses of the association between TG/HDL‐C ratio and unplanned revascularization. Forest plot of multivariable Cox regression (Model 3) showing the association across prespecified subgroups. For each subgroup, the model was adjusted for all Model 3 covariates except the stratifying variable. Interaction was assessed in the overall cohort by adding a multiplicative cross product term to Model 3, and corresponding *p* values are displayed.

Subgroup analyses used Cox models adjusted for the Model 3 covariates, excluding the stratifying variable in each analysis (Figure 3). The positive association between TG/HDL‐C ratio and unplanned revascularization was generally consistent across strata, with most hazard ratios above 1.0. No statistically significant interactions were observed (all *p* for interaction > 0.05), indicating that the strength of the association did not differ materially by baseline characteristics.

In the age‐stratified analysis, the association was stronger in participants aged ≥ 73 years (HR = 1.070; 95% CI, 1.016–1.128; *p* = 0.011) compared with those aged < 73 years (HR = 1.020; 95% CI, 0.991–1.050; *p* = 0.176). Significant associations were also observed in males (HR = 1.030; 95% CI, 1.008–1.035; *p* = 0.008), participants without prior myocardial infarction (HR = 1.033; 95% CI, 1.004–1.063; *p* = 0.025), those with prior PCI (HR = 1.028; 95% CI, 1.004–1.052; *p* = 0.018), and those with two‐vessel disease (HR = 1.110; 95% CI, 1.039–1.188; *p* = 0.002). These findings suggest that while the association was directionally similar across subgroups, TG/HDL‐C ratio may be more readily detectable as a risk factor in certain patient groups.

## 4 Discussion

In this retrospective cohort of elderly patients with type 2 diabetes and established coronary artery disease undergoing angiography, we found that a higher TG/HDL‐C ratio was independently associated with an increased risk of unplanned coronary revascularization over long‐term follow‐up. This relationship remained significant after adjustment for a comprehensive set of demographic, clinical, and laboratory covariates and was confirmed in both continuous and dose–response analyses. The restricted cubic spline model suggested a mildly nonlinear association, with a relatively flat risk profile below approximately 3.427 and a progressively increasing risk thereafter. Subgroup analyses demonstrated that this association was consistent across patient strata, with particularly notable effects in older patients (≥ 73 years), males, those without prior myocardial infarction, those with a history of PCI, and those with two‐vessel disease. These findings are consistent with previous studies showing that lipid‐ and glucose‐related indices, such as the triglyceride‐glucose index, also predict adverse cardiovascular outcomes in elderly diabetic patients [9].

Our findings extend previous work linking TG/HDL‐C ratio with adverse cardiovascular outcomes [10, 11, 19]. The TG/HDL‐C ratio is a simple, reproducible index reflecting an atherogenic lipid profile [20, 21] and insulin resistance [22], both of which play central roles in atherogenesis and plaque progression. Elevated TG levels promote small, dense LDL particles and impair HDL‐mediated reverse cholesterol transport, while reduced HDL‐C limits anti‐inflammatory and antioxidative functions [23, 24, 25].

Recent experimental studies have shown that disturbances in vascular lipid metabolism contribute to endothelial dysfunction and adverse vascular remodeling, providing a mechanistic link between dyslipidemia and plaque vulnerability [26]. Imaging studies have demonstrated that a higher TG/HDL‐C ratio is associated with greater coronary calcification and plaque burden [27, 28]. Calculated small dense LDL‐C has been correlated with soft and vulnerable carotid plaques on CT angiography [29]. Experimental research also shows that disturbances in endothelial triglyceride metabolism can impair endothelial function and promote atherogenesis [30]. Together, these findings suggest that elevated TG may accelerate the formation of sdLDL and endothelial injury, leading to plaque vulnerability and the increased likelihood of repeat PCI observed in our study. Recent advances in intravascular imaging, such as OCT‐guided PCI in elderly patients, have further illustrated how plaque morphology and vulnerability directly influence revascularization outcomes [31]. These mechanisms provide a biologically plausible explanation for the observed relationship between higher TG/HDL‐C ratio and recurrent ischemic events after PCI.

The observed nonlinear pattern suggests that maintaining the TG/HDL‐C ratio below approximately 3.427 may be associated with minimal incremental risk, whereas further increases could confer a disproportionately higher hazard [32, 33]. This threshold is close to that reported in other populations for predicting metabolic syndrome and cardiovascular events [34], underscoring the potential utility of the TG/HDL‐C ratio as a simple, clinically actionable marker for secondary prevention. Importantly, the persistence of the association after full multivariable adjustment indicates that the TG/HDL‐C ratio provides prognostic information beyond traditional lipid measures such as LDL‐C or total cholesterol [35, 36]. Nevertheless, the borderline significance for nonlinearity indicates that this threshold should be interpreted with caution and validated in future research.

Our study has several notable strengths. It specifically targeted an elderly population with type 2 diabetes mellitus and established coronary artery disease, a high‐risk group in whom evidence regarding the prognostic value of TG/HDL‐C ratio is scarce. The large sample size and extended follow‐up period ensured sufficient statistical power to detect clinically meaningful associations. Rigorous statistical approaches, including multivariable‐adjusted Cox regression, restricted cubic spline modeling, and stratified analyses with interaction testing, were applied to enhance robustness and reduce residual confounding. TG/HDL‐C ratio is a simple, cost‐effective, and readily available biomarker derived from routine lipid measurements, making our findings directly translatable to real‐world clinical practice [37]. The identification of a potential risk threshold offers practical implications for individualized secondary prevention strategies.

Several limitations should be acknowledged. The inclusion period spanned more than a decade, during which PCI techniques and adjunctive pharmacotherapy evolved; although we adjusted for key clinical and procedural variables, residual confounding by treatment era cannot be completely excluded. The retrospective, single‐center design may limit generalizability, and selection bias is possible. In addition, the TG/HDL‐C ratio was assessed only at baseline, precluding evaluation of longitudinal changes that may yield additional prognostic insights. Despite these limitations, the consistent results across multiple analytical approaches suggest that they are unlikely to have materially affected the main conclusions.

## 5 Conclusion

In conclusion, in elderly patients with type 2 diabetes and CAD undergoing PCI, a higher TG/HDL‐C ratio was independently associated with an increased risk of unplanned coronary revascularization, with a possible threshold at approximately 3.427. These findings support the potential use of the TG/HDL‐C ratio as a simple, cost‐effective biomarker for long‐term risk stratification in this high‐risk group. Future multicenter studies with serial lipid assessments are warranted to validate these findings and to determine whether interventions targeting the TG/HDL‐C ratio can improve clinical outcomes.

## Author Contributions

Wei Zhu and Yin Zhang contributed to data collection and management. Pan Gao conducted the data analyses and drafted the manuscript. All authors participated in the interpretation of results, revised the manuscript critically for important intellectual content, and approved the final version for submission.

## Funding

This work was funded by the Chongqing Natural Science Foundation under grant CSTB2022NSCQ‐MSX0209, as well as the National Key R&D Program of China, specifically grants 2020YFC2008903.

## Ethics Statement

The Ethics Committee of Southwest Hospital, which is affiliated with Third Military Medical University (Army Medical University, Approval No. (B) KY2021189), approved the study protocol.

## Conflicts of Interest

The authors declare no conflicts of interest.
